# Using tablets to support self-regulated learning in a longitudinal integrated clerkship

**DOI:** 10.3402/meo.v19.23638

**Published:** 2014-03-12

**Authors:** Dylan Archbold Hufty Alegría, Christy Boscardin, Ann Poncelet, Chandler Mayfield, Maria Wamsley

**Affiliations:** 1 Office of Technology Enhanced Learning, School of Medicine, University of California–San Francisco, San Francisco, CA, USA; 2 Research and Development in Medical Education, School of Medicine, University of California–San Francisco, San Francisco, CA, USA; 3 Department of Neurology, University of California–San Francisco, San Francisco, CA, USA; 4 Department of Internal Medicine, University of California–San Francisco, San Francisco, CA, USA

**Keywords:** mobile learning, self-regulated learning, clinical learning, longitudinal integrated clerkship, workplace learning

## Abstract

**Introduction:**

The need to train physicians committed to learning throughout their careers has prompted medical schools to encourage the development and practice of self-regulated learning by students. Longitudinal integrated clerkships (LICs) require students to exercise self-regulated learning skills. As mobile tools, tablets can potentially support self-regulation among LIC students.

**Methods:**

We provided 15 LIC students with tablet computers with access to the electronic health record (EHR), to track their patient cohort, and a multiplatform online notebook, to support documentation and retrieval of self-identified clinical learning issues. Students received a 1-hour workshop on the relevant features of the tablet and online notebook. Two focus groups with the students were used to evaluate the program, one early and one late in the year and were coded by two raters.

**Results:**

Students used the tablet to support their self-regulated learning in ways that were unique to their learning styles and increased access to resources and utilization of down-time. Students who used the tablet to self-monitor and target learning demonstrated the utility of tablets as learning tools.

**Conclusions:**

LICs are environments rich in opportunity for self-regulated learning. Tablets can enhance students’ ability to develop and employ self-regulatory skills in a clinical context.

## Introduction

A call for the training of physicians committed to learning throughout their careers has led to a push for development of self-regulated learning skills during medical school (1–4). Zimmerman defined self-regulated learning as the use of self-monitoring and self-control while learning and an internal locus of motivation and ongoing self-critique of both learning and learning processes (5, 6). Efforts to increase self-regulated learning at all levels of undergraduate medical education have shown moderate success (2, 3, 7, 8). With increased interest in self-regulated learning, a recent reflective analysis by Brydges and Butler challenges the medical education research community to extend our efforts to identify best practices that support and facilitate self-regulation (2).

Tablet computers could enhance students’ ability to self-evaluate, plan learning, and pursue their educational needs. These tools are rapidly permeating both the clinical and educational environments. While literature describing uses for tablets during the pre-clinical years has emerged, few studies focused on how students use these tools to drive learning in the clinical years (9–11). Evidence from the use of personal digital assistants (PDAs) suggest that tablets may facilitate self-regulated learning by allowing students to make use of wasted time, to consolidate learning through repetition as well as to schedule and plan learning activities (12, 13).

Interest in longitudinal integrated clerkships (LICs) has increased in recent years predicated on the perspective that these year-long clinical experiences provide opportunities for genuine workplace experiences; continuity of relationships with patients and supervising doctors; and self-regulated learning (14, 15). LIC students must balance their learning needs in several specialties and continually monitor their learning progress. Students in LICs express the need to independently identify and correct learning gaps and rely on internal drive for knowledge, a hallmark of self-regulated learning (16). The central role of self-regulated learning in an LIC makes it an ideal context in which to evaluate tools such as tablet computers that may help facilitate and scaffold self-regulated learning.

The manner in which students use tablet computers on clinical rotations remains undefined. To better understand these tools and plan how they might be better employed, an exploratory qualitative approach was undertaken. This study seeks to describe the manner in which students in an LIC employ tablet computers for self-regulated learning.

## Methods

### Setting and participants

The Parnassus Integrated Structured Clinical Experiences (PISCES) program is a year-long LIC at the major tertiary hospital at UCSF (17). Third-year medical students are assigned to a faculty or resident preceptor for each core discipline and 15 students were enrolled during the 2012–2013 academic year. Faculty and resident preceptors participate on a volunteer basis. Students’ schedules are divided into half-day outpatient experiences with their clinical preceptors. Students also spend time in the emergency department and inpatient surgery and medicine. There is a weekly half-day of didactics. Student self-regulatory skills are supported by clerkship learning objectives and self-developed quarterly individualized learning plans. Students create learning issues, brief clinically focused research reports, to demonstrate their learning to preceptors (17). The University of California, San Francisco (UCSF) Institutional Review Board, approved this study and all participants voluntarily consented to be included in the study.

### Intervention

Students were provided with an Apple iPad 2 WiFi-model with 16 GB of storage and an Evernote premium account. Evernote is a multiplatform journaling application with sharing features. Students received a 1-hour workshop on the relevant features of both the iPad and Evernote, including instruction on mobile access to the EHR and the use of Evernote to document and retrieve learning issues.

### Data collection

All 15 study participants were invited in person and by email to attend two 1-hour long focus groups, one 3 months into the clerkship and one 3 months prior to the end of the clerkship to understand changes in the use over the course of the year.

### Focus groups

Focus group protocols were developed to elucidate patterns of tablet use and elicit reflection on the impact of the intervention on self-regulation. Focus group questions directly targeted the three major components of self-regulation: use of self-regulatory learning strategies, repeated self-evaluation and intrinsic motivation (Appendix). Both sessions were conducted by a moderator (PN) and assistant moderator (DA) and were recorded and transcribed for analysis. The assistant moderator recorded qualitative impressions of group dynamics and body language. Themes and individual perspectives were extracted by two independent raters (MW, DA) using qualitative micro-interlocutor analysis and discrepant ratings were reconciled. This method incorporates semi-quantitative data, relationships and non-verbal cues in the analysis of focus group data (18). These themes were mapped to a pre-conceived framework for the evaluation of technological interventions, which was derived from previously published work (Table 1, 19).

**Table 1:** Focus group analysis framework

Patient care	Learning	Experience factors	Impact factors
Content consumption • Patient info at the POC* • Reference info at POC* • Remote access to patient info Content creation • Taking notes with patient • Building note templates Content sharing • Patient education • Oral Presentation	Content consumption • Reading texts • Revisiting learning issues (LIs) Content creation • Documentation of LIs • Notes during lecture Monitoring learning Content sharing • Demonstrate learning to faculty • Study groups	Alternatives Resources Accessibility • Contexts • Portability • Accessories Perceptions Variance	Cost Change over time

* POC, point of care.

## Results

### Participation

The first and second focus groups were attended by 11/15 (73%) and 8/15 (53%) students, respectively.

#### Patient care

Most students in the focus groups did not use the tablet to access information during face-to-face interactions with patients.

… if I'm at a clinic, and there's a busy schedule and I'm seeing patients, [the tablet is] not part of how I see patients, it's not part of how I chart, so … I'm not using it at all.

In contrast, one of the students who self-identified as a technophile used the tablet as a tool to increase efficiency in clinic.

Within the theme of patient care, students had general aspirational goals of accessing patient information at the point of care but generally preferred computers when available. However, a subset of students did use remote access to the EHR on the tablet to access patient records while away from the clinic. A minority of students reported using the tablet to access reference information at the point of care.

… if I go to the operating room we can go over the anatomy of where we're going today.

One student took handwritten notes during patient encounters on the tablet but other students did not record clinical data on the tablet during encounters. Students generally did not find the tablet to be a useful tool for presenting patient information to their preceptors because they did not tend to take notes on their devices.

Several students expressed interest in using the tablet to counsel patients, and suggested a number of resources to accomplish that goal. However, they universally acknowledged that student-driven patient education is not always supported in clinic.

At this level we don't do that or even explicitly are requested not to because if we get it wrong our preceptor has to backtrack.

#### Learning

The tablet was used in a variety of ways to support learning. Students universally engaged in reading content on their tablets whether at home or in clinic and to study for standardized assessments. A subset of students also used the tablet to collect their own learning resources.

Students found the tablet to be valuable as a tool to read textbooks. They reported that the tablet was more portable than the numerous reference books that they required on a weekly basis as they switched between clinics. Additionally, a majority of students strongly preferred reading on a tablet to a smart phone.

I would never bring up a PubMed article … and read it in its entirety on my phone … and I do that all the time on my [tablet].

Most students reported that they appreciated the ability to access learning issues quickly on their mobile devices. Half of the students almost never revisited learning issues and the other half revisited them rarely. Most students used email or text documents for learning issues. One student used Evernote to track, capture and review learning issues on a daily basis. Universally, students wished that they had taken notes using the tablet during the preclinical years so that they could access those notes easily from clinic.

While most students endorsed reading prior learning issues on the tablet, few used the tablet to create learning issues. One student's statement captured this sentiment; ‘I like making my learning issues on a computer and then transferring them [to the tablet]’. Approximately half of students used the tablet to take notes during inpatient or small group didactic sessions.

In general, students were enthusiastic about accessibility of banks of practice questions for national tests on the tablet as a way to assess and track their learning. They used these tools to determine what they needed to study next. Beyond this, most students did not use the tablet to track their knowledge with the exception of the one student who used Evernote to track her learning saying, ‘it's helped me stay more organized with my learning … to keep things in one place and a running tab of what I've studied and what I want to study’.

A group of students found the tablet to be a useful tool to share their knowledge. More than half of the students used the tablet to demonstrate to faculty that they were learning, either by sending the faculty a learning issue that they had created or by presenting a learning issue using the tablet as a tool. They significantly preferred the tablet to a smart phone. Only one student had considered using the tablet as a collaborative tool during group study.

#### Experience factors

Students were initially vocal about their concerns regarding the usability and portability of the tablet. But these concerns lessened over the course of the year-long clerkship.

These concerns stemmed largely from difficulty with the virtual keyboard and with size and portability concerns. Students had variable opinions regarding portability. On one hand, students thought that a smaller device would be more convenient.

[I would keep the tablet] in my white coat, and now I only wear my white coat at one clinic [so I do not carry it anymore].

Alternatively students all agreed with the statement, ‘One of the big limitations of the [tablets] … is that they get stolen’. A group of students stopped using the tablet as a result of these barriers. One tablet was broken during the year and none were stolen.

Students were also concerned that their preceptors would think that they were ‘texting or doing email’ but also said that using the tablet was more likely to be perceived as work than using a smart phone.

Tablets were used throughout the hospital, in clinic, and at home. Students thought that the tablet would be of particular use as a laptop replacement during lectures. In clinic it had the greatest value, ‘when [clinical] activities are lacking and there is more downtime’. In general the students agreed with the sentiment voiced by one of the most active users.

[It's] very valuable in having the [tablet] just there, ready to do some work, like reading, and not necessarily having a computer near by … I think that's nice and empowering.

#### Impact factors

Student opinions of factors that might affect the implementation of a tablet program in a larger audience, such as cost, were mixed. Most students acknowledged the expense of the tablet to the school, saying ‘It's a pretty expensive cost for students … especially because most of our money is in loans’. Students who identified as more technologically savvy pointed out that the expense was reduced if all reading was done on the device, ‘It would have paid for itself [not having to print] the syllabus’.

## Discussion

The use of tablets among students in our study was very much in line with prior descriptions of PDA and smartphone use in traditional block clerkships (12, 20). Overall, students perceived more value in the use of the tablet as an educational tool compared to its value for use as a clinical tool. Students identified the tablet as an effective way to access medical resources that were otherwise unavailable and were more likely to access full research articles on tablets versus smartphones. In this way, the tablet supported their self-regulated learning and reduced barriers to the pursuit of internally motivated learning. A subset of students used the tablet to track their educational progress using question banks and through the accumulation of learning issues. This behavior typifies the self-monitoring and evaluation elements of self-regulated learning. Taken together, our results support overall student satisfaction with tablets as a tool to support increased availability of learning materials and development of self-regulated learning during the clinical years.

Tablets were of less use to students as clinical tools. Although gains in clinical efficiency have been published among resident physicians (21), students in our cohort largely did not feel that the tablet added to their clinical workflow. This is likely related to the different responsibilities of residents and third-year medical students. The intervention was also limited by challenges with carrying and inputting learning data into the device and a subset of students stopped using the device in a clinical context as a consequence of these frustrations. These challenges may be addressed as tablet technology is further developed.

The limitations of this study include the small sample size and lack of a control group. However, the majority of LIC students participated in focus groups and our sample is thus representative of the group. Although this is a single institution study, we believe that the results are generalizable to other LICs with similar structures. These results may potentially also be extended to traditional block clerkships as the needs of students in these clerkships increasingly mirror the needs of our cohort. The creation of learning issues and the development of self-regulated learning skills are also likely to benefit traditional block clerkship students. Moreover, as early preclinical workplace-based experiences become a more important part of medical education, preclinical medical students are likely to begin to resemble this longitudinal cohort in their need for workplace learning skills (22).

## Conclusion

Self-regulated learning will remain a critical skill to encourage and develop in medical students. This study demonstrates that patterns of tablet use among students in a LIC support self-regulated learning in a way unique to each individual's learning style. Motivated students were able to significantly enhance their learning process by taking full advantage of the technology and all students used the tablet to foster self-regulatory skills. Additional research of tablets in the clinical years should focus on outcome and variables or surrogates that have been demonstrated to correlate with improved learning. Other emerging technologies are likely to change the way that students learn. Further research is needed to better define pathways to integrating unexpected and potentially useful technology into medical education in a timely and thoughtful manner.

## Appendix

Focus Group Quarter 1 Questions

1. During the first 3 months of PISCES how have you used the iPad?

 a. PROBE: If no one volunteers information, start round robin.

 b. PROBE: When do you use it?

 c. PROBE: Where do you use it?

2. How do you capture, access, and share your learning issues?

 a. PROBE: Are you using Evernote?

 b. PROBE: If not what do you use to capture learning issues?

 c. PROBE: Do you write learning issues on the iPad? (ask even if not using Evernote)

 d. PROBE: If so where do you write them (clinic, bus, home)?

 e. PROBE: Do you access learning issues on the iPad?

 f. PROBE: How do you access them?

3. How do you access the EMR?

 a. PROBE: Do you access it on the iPad?

 b. PROBE: If yes, do you use Canto or Citrix?

 c. PROBE: Where do you access the EMR using the iPad?

 d. PROBE: Any thoughts on use during your acute care sessions (ED/SACC)?

4. What Apps and Websites do you use most frequently on the iPad?

 a. PROBE: When do you use them?

 b. PROBE: How do they work together?

 c. PROBE: How do they work with clinic computers or your home computer?

5. What can UCSF do to improve the experience of students using iPads during the clinical years?

 a. PROBE: Tell me how it went for you during your surgery rotation/ED shift/clinic session.

 b. PROBE: How have you carried the iPad around?

 c. PROBE: Are there any times that you do not bring the iPad?

 d. PROBE: What are faculty/resident/fellow students to the iPad?

 e. PROBE: Did you feel that there was adequate tech support?

 f. PROBE: Were there interface or compatibility issues that you experienced?

 g. PROBE: What things worked well?

6. Do you use other mobile devices for clinical learning (laptop, smartphone)?

 a. PROBE: If yes, when do you prefer the iPad to a smartphone or laptop?

 b. PROBE: If yes, when do you prefer a smartphone or laptop to the iPad?

7. Comparing your experience to peers who either do not have or do not use an iPad would you recommend that rising MS3s purchase an iPad for school?

 a. PROBE: Why or why not?

 Focus Group Quarter 3 Questions

8. During PISCES how have you used the iPad?

 a. Ask for a show of hands of who is using the iPad for clinical learning?

 b. PROBE: If no one volunteers information, start round robin.

 c. PROBE: How has your use changed over the year?

 d. PROBE: What surprised you about how you are using it now?

9. How are you capturing/sharing and accessing your learning issues now?

 a. Ask for a show of hands of who is still using Evernote.

 b. PROBE: If you are not using Evernote what do you use to capture learning issues?

 c. PROBE: How has your behavior changed during the year regarding learning issues?

 d. PROBE: If you changed the way you create and access learning issues why did you make the change?

 e. PROBE: Are you sharing learning issues? In what settings?

 f. PROBE: Do you revisit learning issues that you wrote earlier in the year? When?

10. How has the iPad impacted your ability to monitor and evaluate your learning? Or has it had no impact?

 a. PROBE: How has it enhanced or detracted from your appreciation of what you already know?

 b. PROBE: How has it enhanced or detracted from your ability to assess what you do not know?

 c. PROBE: How has it enhanced or detracted from your ability to appropriately choose the next step in your learning?

 d. PROBE: What features of the iPad was most helpful in evaluating and monitoring your knowledge?

11. Has the iPad given changed your sense of ownership over your learning?

 a. *If students do not understand the question*: Are you learning things that YOU want to learn, when you want to learn them?

 b. PROBE: What aspects of the iPad fostered that sense?

 c. PROBE: If no, what do you think might enhance ownership in your learning?

12. Has the iPad helped you develop learning strategies that you think you can use during 4th year, residency and as a practicing clinician?

 a. PROBE: If yes, in what ways and what aspects of the iPad fostered that sense?

 b. PROBE: If no, what if any technological solutions might assist you in developing those skills?

13. What Apps and Websites do you use now that you were not using earlier in the year?

 a. PROBE: When do you use them?

 b. PROBE: How do they work together?

 c. PROBE: How do they work with clinic computers or your home computer?

14. What can UCSF do to improve the experience of students using iPads during the clinical years?

 a. PROBE: What things worked well?

 b. PROBE: What things did not work well?

15. Comparing your experience to peers who either do not have or do not use an iPad would you recommend that rising MS3s purchase an iPad for school?

 a. PROBE: Why or why not?
